# High-Throughput Screening of *Chlorella Vulgaris* Growth Kinetics inside a Droplet-Based Microfluidic Device under Irradiance and Nitrate Stress Conditions

**DOI:** 10.3390/biom9070276

**Published:** 2019-07-12

**Authors:** Marwa Gamal Saad, Noura Sayed Dosoky, Muhammad Shuja Khan, Mohamed Shafick Zoromba, Laila Mekki, Magdy El-Bana, David Nobles, Hesham Mohamed Shafik

**Affiliations:** 1 Department of Electrical and Computer Engineering, Texas A&M University, College Station, TX 77843, USA; 2 Department of Botany, Faculty of Science, Port Said University, Port Said 42521, Egypt; 3 Department of Chemistry, University of Alabama in Huntsville, Huntsville, AL 35899, USA; 4 Department of Electrical and Computer Engineering, University of Alabama in Huntsville, Huntsville, AL 35899, USA; 5 Chemical and Materials Engineering Department, King Abdulaziz University, Rabigh 21911, Saudi Arabia; 6 Department of Chemistry, Faculty of Science, Port-Said University, Port Said 42521, Egypt; 7 Department of Botany, Faculty of Science, Suez Canal University, Ismailia, Egypt; 8 UTEX Culture Collection of Algae, College of Natural Sciences, University of Texas at Austin, Austin, TX 78712, USA

**Keywords:** *Chlorella vulgaris*, biodiesel, droplet-based microfluidic device, conventional batch cultures, light, nitrate, biomass productivity

## Abstract

Biodiesel is an eco-friendly renewable fuel that can be derived from microalgae. Maximization of biomass and lipid productivities are considered the main challenges for algal biodiesel production. Since conventional batch cultures are time-, space-, and reagent-consuming with many restrictions to apply many replicates, microfluidic technology has recently emerged as an alternative low-cost and efficient technology with high throughput repeatability and reproducibility. Different applications of microfluidic devices in algal biotechnology have been reported, including cell identification, sorting, trapping, and metabolic screening. In this work, *Chlorella vulgaris* was investigated by encapsulating in a simple droplet-based micro-array device at different light intensities of 20, 80, and 200 µmol/m^2^/s combined with different nitrate concentrations of 17.6, 8.8, and 4.4 mM. The growth results for *C. vulgaris* within microfluidic device were compared to the conventional batch culture method. In addition, the effect of combined stress of deficiencies in irradiance and nitrogen availability were studied to illustrate their impact on the metabolic profiling of microalgae. The results showed that the most optimum favorable culturing conditions for *Chlorella vulgaris* growth within the microfluidic channels were 17.6 mM and 80 µmol/m^2^/s.

## 1. Introduction

Microalgae are photosynthetic organisms and a promising feedstock for different products, such as antibiotics, cosmetics, toothpastes, alginates, agar [1,2,3], and renewable fuels, e.g., biodiesel, biohydrogen, and bioethanol [4,5], because they conserve water and land resources [6]. They can also be used as biofertilizers and bioremediators [7]. The history of using algae as a renewable energy feedstock dates back to the 1970s [4]. The main challenges for algae production are maximizing biomass and lipid productivities by optimizing culture and production conditions which are considered to be time and labor extensive. Different factors affect biomass and the behavior of algal cells [8]. These factors are species-specific and can be categorized into three groups: biological, chemical, and physical factors. Biological factors are species size, shape, age, and cell composition, while chemical factors include nutrients concentrations and availability, C:N and N:P ratios, and the physical factors cover light, temperature, and pH [9]. Testing multiple growth factors simultaneously provides multidimensional information on factors affecting the growth. Among those, photon flux and nitrogen availability are important factors that influence algal growth and its behavior over a period of time.

*Chlorella vulgaris (C. vulgaris)* is a eukaryotic unicellular chlorophyte [10]. It has been cultured for various commercial applications, like antioxidants, human nutrition, and bioremediation [11,12,13]. *C. vulgaris* was also cultured as a model strain for biofuel production in the U.S. and Europe [6]. *Further*, *C. vulgaris* can adapt to extreme conditions, such as 10–15% CO_2_ vol. % [14], high temperature [15], acidic pH [16], and up to 200 and 50 ppm of NO and SO_2_, respectively [14]. Traditional batch cultures have been used previously which provide appropriate biomass for fatty acid profiles, proteins, and carbohydrate analyses. However, they exhibit numerous disadvantages, such as limited numbers of replicates, restricted limits of testing factors, and are space-, reagent-, and time-consuming [17]. On the other hand, there has been tremendous interest recently to utilize microfluidic devices as a tool to investigate the microalgae under different conditions.

*C. vulgaris* has been tested inside microfluidics with different designs for a variety of purposes [18,19,20,21,22]. A microfluidic device (lab-on-a-chip) offers the capability to manipulate a very small volume of fluids; 10^−6^ to 10^−18^ L through connected microchannels with dimensions from 10 to 100 µm [23]. This reveals tremendous miniaturization capabilities and enables innovative ways of performing biotests without the need for conventional laboratory infrastructure [5]. Poly dimethylsiloxane (PDMS) is the most popular polymer used for device fabrication especially for biological and chemical samples. PDMS is the most popular polymer used for device modeling because it is inexpensive, transparent, and biocompatible [23,24]. It further prevents solution evaporation for a certain time [25], and is more appropriate for analyses of biological and chemical samples than glass and silicon with faster prototyping and the possibility of electrosensing [26].

In this work, a microfluidic device capable of simultaneously testing different culture conditions using a single system design is presented. The design is based on creating separated channels parallel to each other. Each of these channels can be loaded with a different solution of nitrate concentration of 17.6, 8.8, and 4.4 mM. In addition, we sought to determine the feasibility of using the device as a monitoring tool in place of conventional batch techniques. We characterized the relationship between the availability of light and nitrate with the growth of *C. vulgaris* cells using both conventional culturing techniques and microfluidic techniques.

## 2. Materials and Methods

### 2.1. Droplet-Based Microfluidic Design

**Figure 1:**
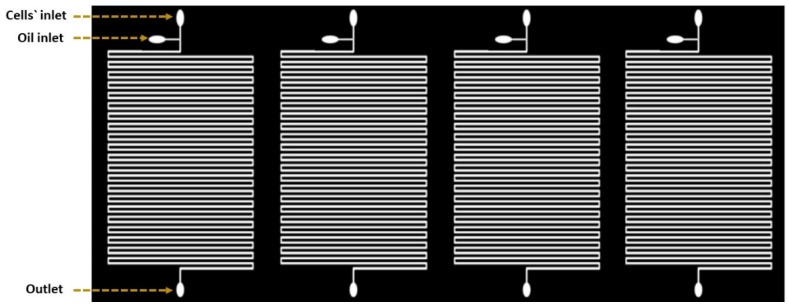
Schematic diagram of the microfluidic device design. The design is based on creating separated channels parallel to each other. Each of these channels can be loaded with a different solution.

The droplet-based device was consisting of four parallel microfluidic devices (Figure 1). Each device was comprised of serpentine channels which were separated by a distance of about 160 µm. Four devices were located beside each other horizontally and the samples were processed simultaneously with constant flow rate. Inputs were used to facilitate droplet generation with one output for the waste. The two inputs were presented as two inlet channels: a 200 µm wide channel for the carrier oil and a 160 µm wide channel for the cell suspension. Channels had narrower width compared to the utilized droplet size to slightly squeeze the droplets to maintain consistent space between neighboring droplets. This provides precise droplet ordering for time-course growth analysis of the same droplet, and further prevents unexpected droplet merging throughout the culturing period [18].

### 2.2. Device Fabrication

The design of the device was performed using SOLIDWORKS^®^ software (SolidWorks Corp., Waltham, MA, USA). The design was patterned on a silicon wafer surface with SU-8 photoresist (SU-8 2075, Microchem Corp., Westborough, MA, USA) using a conventional photolithography process then coated with the surfactant (tridecafluoro-1,1,2,2-tetrahydrooctyl) trichlorosilane (United Chemical Technologies, Inc., Bristol, PA, USA) to facilitate PDMS release from the master molds. Afterwards, the fabrication process ended with PDMS molding. PDMS layer with a thickness of 4 mm were bonded with a glass slide of 50.8 × 9.0 × 76.2 mm by oxygen plasma treatment. After assembly, PDMS devices were loaded with Aquapel (Pittsburgh Glass Works LLC., Pittsburgh, PA, USA) to generate hydrophobic properties inside channels’ surfaces to stabilize droplets.

### 2.3. Species Preparation

A freshwater *C*. *vulgaris* was isolated from Port Said, Egypt. The species was purified on a solidified BG11 medium [27]. *C*. *vulgaris* was examined for early identification using binocular light microscope (SME-F4D, Rating: 85 V to 265 V, 50/60 Hz, Halogen lamp: 60 V 20 W, delay-action fuse: 1 A). Axenic culture was prepared using antibiotic assessment technique. One capsule of Chloramphenicol was dissolved in 10 mL 70% ethanol (capsule, MEHTA, India) then added to the culture which left to grow for two days then samples were streaked on nutrient agar medium for the confirmation of culture free-bacteria. Axenic colonies were cultured in 10 mL BG11 medium for five days at 20 °C and 80 ± 0.13 µmol/m^2^/s with continuous lighting. The culture volume was gradually increased per two weeks by 10% (*v*/*v*) to maintain cells. These cultures were used as stock cultures for next experiments. All steps were done under aseptic conditions. Purified samples were examined using Olympus^®^ microscope with magnification power of 400×.

### 2.4. Species Identification

**Figure 2:**
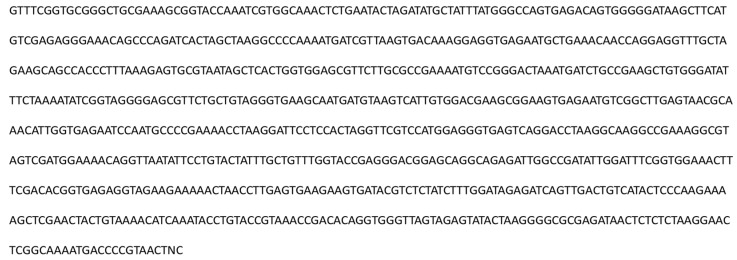
The 23S rRNA gene of *Chlorella vulgaris* sequences in the NCBI using GeneDoc. The isolated strain was identified as *Chlorella vulgaris* according to the morphological characterization and molecular methods. The Basic Local Alignment Search Tool (BLAST) result of the amplified sequence with other *Chlorella* strains in The National Center for Biotechnology Information (NCBI) showed 98% sequence similarity to the 23S small subunit rRNA of *Chlorella* species.

*C*. *vulgaris* was identified using the 23S rRNA gene. Genomic DNA was purified as previously described [28]. The quality and quantity of purified DNA was assessed using a NanoDrop^®^ (ND-1000 UV-Vis Spectrophotometer, USA). The 23s rDNA was amplified by PCR, as described previously [29], using Platinum Taq DNA polymerase (2700 Applied Biosystems, Gene Amp^®^, Singapore) and 40–100 ng of genomic DNA per 100 µl reaction. PCR conditions were as follows: initial denaturation at 94 °C for 2 min, followed by 40 cycles at 94 °C for 30 s, 55 °C for 1 min, and 72 °C for 2 min with a final extension at 72 °C for 7 min followed by a hold at 4 °C. Two 23S rDNA primers were included, 23U1 (5′-AGG GGT AAA ACA CTA TTT CG-3′) and 23U2 (5′-CCT TCT CCC GAA GTT ACG-3′) [29]. Sanger DNA sequencing was performed by the University of Texas DNA sequencing facility using Applied Biosystems 3730/3730XL DNA Analyzers and BigDye Terminator v3.1 chemistry according to facility protocols. Bioinformatics analysis of the amplified sequence was performed by comparing similar sequences on the NCBI database via BLAST (Figure 2).

### 2.5. Droplet Generation

Cell suspension of *C. vulgaris* with concentration of 2002 ± 20.3 × 10^4^ cells/ mL was loaded to 1 mL syringe. The carrier oil; 3M™ Fluorinert™ Liquid FC-40 oil (Maplewood, MN, USA) with 2.5% surfactant (008-FluoroSurfactant, Ran Biotechnologies, MA, USA) was loaded to another 1 mL syringe. Syringes were connected separately to the channels’ inlets using tubing. The flow of fluids inside syringes were automatically controlled using Fusion Touch 400 pump (Chemyx Inc., Stafford, TX, USA). As described previously [30], the 270-µm droplets were generated by consistently flowing cell suspension and oil with flow rates of 150 and 300 µL/h, respectively [31]. After 5 min, approximately 100 uniform droplets were captured inside each channel. Most droplets had one to three cells. Droplets maintained in that device without reported merging issues for monitoring growth. Each channel in the device was loaded with different sodium nitrate concentration of 17.6, 8.8, and 4.4 mM. After loading all channels with samples, different devices were subjected to different light intensity of 20 ± 0.31, 80 ± 0.37, and 200 ± 0.39 µmol/m^2^/s using LED lamps with 100w and 6500 K at 20 °C. 

### 2.6. Traditional Batch Cultures

Inoculations were prepared by adding a biomass concentration of 1128 ± 24 × 10^4^ cell/mL to 10 mL of BG11 medium. BG11 medium with different sodium nitrate concentrations of 17.6, 8.8, and 4.4 mM were examined. Each set of nitrate cultures was tested under different light intensity of 20.88 ± 1, 80.17 ± 1, and 200.4 ± 1 µmol/m^2^/s at 20 °C.

### 2.7. Growth Assay Inside Droplets

Tracking the growth of *C*. *vulgaris* cells encapsulated inside the same droplet in the culture channels allowed a time-course growth analysis, where the growth of cells was characterized by chlorophyll autofluorescence intensity per unit area. At scheduled analysis periods, each device was moved to the Zeiss Axio Observer Z1 microscope (Carl Zeiss MicroImaging, LLC) stage to be imaged separately. The microscope was equipped with a digital camera (Orca Flash2.8 CMOS Camera) and a filter set (excitation: 460–500 nm, emission > 600 nm). The tiles were set and marked to locate each channel during imaging. The lenses were set on powers of 10 × 5.

The resulted fluorescent intensity of cells was analyzed using ImageJ software [31]. The maximum growth rate was calculated using the following equation [32]:

µ (d^−1^) = ln(N2 − N1)/T2 − T1 (1)

where N2 andN1 are the chlorophyll intensity at the times t2 and t1, respectively. The linear relationship between the cell number/droplet (count/mL) and the relative fluorescent intensity was investigated. Cell number/relative fluorescent intensity equations are as follows:

Y = 5.594X R^2^ = 0.9949 (2)

where Y is the relative fluorescent intensity (a.u.) and X is the cell number/droplet (count/mL). The results are presented as the cell number per droplet for 10 replicates and standard deviation was considered.

### 2.8. Growth Assay for Traditional Batch Cultures

At constant times, cultures were well-shaken then 10 µL of representative samples were loaded in a hemocytometer and counted in four 16 × 16 squares using Nikon light microscopy [33]. Then the summation of cells was multiplied by 10^4^ to estimate the cell number per mL. Two replicates were counted for each sample and the standard deviation were considered.

## 3. Results and Discussion

Nitrogen is important for DNA, protein, enzyme, and membrane synthesis. Nitrate (NO_3_^−^) and ammonia (NH_4_+) are the most common nitrogen forms which algae can uptake [34]. The impact of the nitrogen regime on the growth and fatty acids profiles of *Desmodesmus quadricaudatus* and *Chlorella* sp. has been studied [35]. Further, the growth of *C. vulgaris* has also been significantly enhanced with an increase in light intensity as reported elsewhere [36,37,38]. There are a few other reports published on the metabolic profiling of microalgae cultivated under the combined stress of deficiencies in irradiance and nitrogen availability [39]. Optimum cultivation and production conditions are species-specific [40].

A simple droplet-based platform was used to simultaneously investigate the effect of different culture conditions (light intensity and nitrate availability) on the growth of *C. vulgaris.* The chlorophyll autofluorescence was initially induced using pulse-amplitude-modulating fluorometry, then correlated to carbon dioxide assimilation [41]. It is based on the energy of the absorbed photon by the chlorophyll molecule. That energy will be either used to drive the electron transfer in photosystem II with an opened reaction center or to excite the fluorescence under low light intensities. With increasing the light intensity, the reaction center of photosystem II will close, the electron transfer will cease, and the fluorescence will rise. Relationship between light over time (A) and relation between uniform area (cm^2^) and light intensity (µmol/m^2^/s) (B) are shown in Figure S1. Therefore, the observation of chlorophyll autofluorescence’s induction with its relationship to reactions’ efficiency inside photosystem II provides a rapid and non-destructive method to quantify the quantum yield of photosynthesis [42].

**Figure 3:**
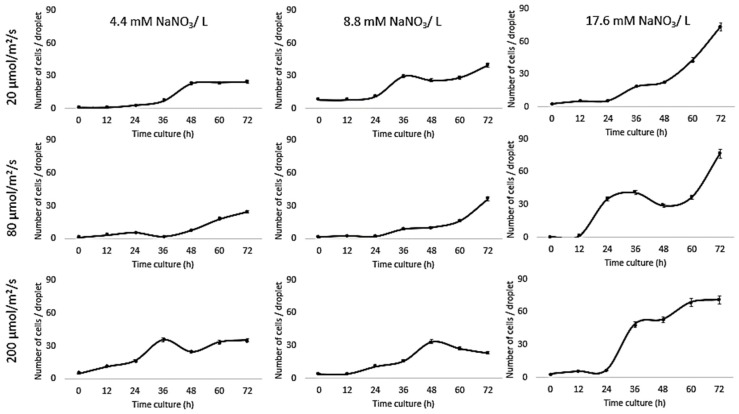
Droplet-based microfluidic technique. Growth kinetics as cell number (count/droplets) for *C*. *vulgaris* cells suspended in BG11 medium with different nitrate concentrations (4.4, 8.8, and 17.6 mM) inside 270 µm droplets for 3 days at 20 °C and different light intensities (20, 80, and 200 µmol/m^2^/s). Standard deviation was applied (n = 10).

**Figure 4:**
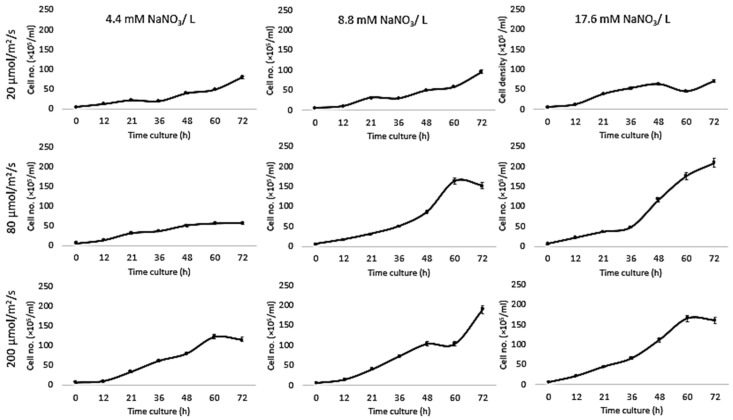
Traditional culture results. Growth kinetics as cell number (count / droplets) for *C*. *vulgaris* cells suspended in BG11 medium with different nitrate concentrations (4.4, 8.8, and 17.6 mM) for 3three days at 20 °C and different light intensities (20, 80, and 200 µmol/m^2^/s). Standard deviation was applied (n = 2).

Growth of *Chlorella vulgaris* was observed over the period of time at different light intensities (20, 80, and 200 µmol/m^2^/s) and different nitrate conditions (17.6, 8.8, and 4.4 mM) using both the conventional batch cultures (Figure 3) and the microfluidic device (Figure 4). The results for *C. vulgaris* culture growth was significant in both techniques within three days of cultivation. The growth was increased with an increase in the concentration of the nitrate and the light intensity. However, the growth behavior of *C. vulgaris* suspended in the regular batch culture showed that the growth was limited at 20 µmol/m^2^/s for all nitrate concentrations, whereas 80 µmol/m^2^/s was reported to be the optimum light intensity conditions for *C. vulgaris* growth. Experimental data shown in Figure 3 were collected up to 72 h and *C. vulgaris* suspended in BG11 medium inside 270 µm droplets did not show being static with in the microfluidic channels. However, these experiments could be extended for a longer period of time if required as the microdroplets can also be used for a longer time period for continuous monitoring.

Based on the experimental evidence presented in this work, the most optimum favorable culturing conditions for *Chlorella vulgaris* growth within the microfluidic channels were 17.6 mM and 80 µmol/m^2^/s. These results are consistent with previous reports where the decreased growth of *C. vulgaris* has been reported at the lower nitrate concentration [10,43,44,45]. On the other hand, no significant decrease in *C. vulgaris* growth was reported with decreasing nitrate concentration [15]. To further optimize the effect of nitrate concentration on *C. vulgaris* growth rate, Jeanfils et al. established a limitation of linear relationship of the C. *vulgaris* growth rate with respect to the nitrate concentration [46].

Our results are in agreement with Kim et al., [47] who tested the influence of light intensity on *Botryococcus braunii* growth in a microfluidic device and found a 1.8-fold increase compared to bulk cultures. Moreover, our comparison investigations concurred with Dewan et al., [48] who investigated the growth kinetics of the microalgae, *C. vulgaris*, in immobilized arrays of nanoliter-scale microfluidic drops and found that the growth rates inside droplets were higher than bulk-scale experiments in most cases. Sung et al., reported the photoautotrophic growth of *C. vulgaris* after culturing cells for 120 h in a droplet-based photobioreactor; a round micropillar array. The growth kinetics were tested under different CO_2_ concentrations (1%, 2.5%, 5%, and 7.5%) and light intensities (35, 55, 100, 150, and 200 μmol/m^2^/s). The growth in a microdroplet showed better cell growth performance compared to a flask culture due to the reduced shading effects and improved mass transfer [49].

Our results are different from those reported by Kim et al., [50] where on-chip results were consistent with bulk culture results for *Chlamydomonas reinhardtii* cultured in an n-hexane-compatible lab-on-a-disc under nitrogen, acetic acid, and iron depletion. Kuntanawat et al. [51] studied the same growth habit of *Spirulina platensis* cultivated in both culturing systems. *Chlamydomonas reinhardtii*, *Chlorella vulgaris*, and *Dunaliella tertiolecta* growth were compared in a simple droplet-based microfluidic system and bulk cultures for 10 days. However, cell viability in the microfluidic device was lower than bulk cultures, and that the doubling time of microalgae grown in microdroplets was similar to growth in bulk [52]. *C. vulgaris* has been encapsulated inside 26 µm droplets with alginate hydrogel microcapsules in a microwell array that incorporated a microbridge structure. A comparison of growth results with those of off-chip cultures demonstrated similar outcomes [21]. Our results were also different from Perin et al. [53], where microfluidic results were 10-fold lower than bulk cultures as a result of culturing *Nannochloropsis gaditana* under different light intensities (6, 60, or 360 μmol/m^2^/s).

**Figure 5:**
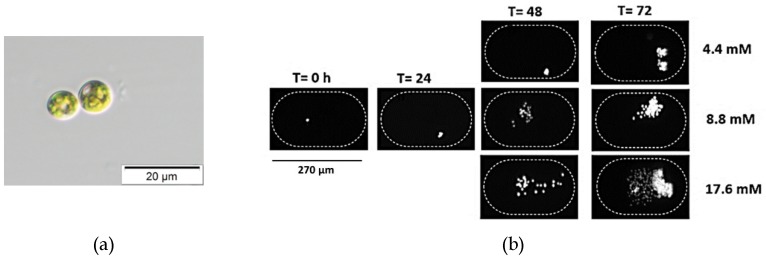
*Chlorella vulgaris* is a unicellular eukaryotic alga, spherical-shaped with a diameter of 3.32 ± 0.99 µm. It has a single cup-shaped chloroplast. (**a**) Bright field image of *C. vulgaris.* (**b**) Time-lapse images of *Chlorella vulgaris* cells inside the microfluidic chambers at time T = 0, 24, 48, and 72 h after inoculation to show growth at 20 °C, 20 µmol/m^2^/s, and different nitrate concentrations (4.4, 8.8, and 17.6 mM). All these experiments were performed from the same inoculant over the same time period.

Since the growth rates of photoautotrophic organisms are highly dependent on the light intensity, comparing data for culturing systems indicated that the higher the nitrate concentration, the higher the growth rate under all tested light intensities (Table 1), whereas the growth inside the traditional system was higher than the droplet cultures due to the availability of the medium. All these experiments were performed from the same inoculant over the same time period (Figure 5).

## 4. Conclusions

A microfluidic device was used as a low-cost, simple, and efficient solution for in vitro experiments to study the effects of different light intensities in combination with varying nitrogen availability on the growth rate of *Chlorella vulgaris*. The growth rates of photoautotrophic organisms are highly dependent on the light intensity. Comparing data for culturing systems indicated that the higher the nitrate concentration, the higher the growth rate under all tested light intensities, whereas the growth inside the conventional system was higher than the droplet cultures due to the availability of the medium. Based on the experimental evidence presented in this work, the most optimum favorable culturing conditions for *Chlorella vulgaris* growth within the microfluidic channels were 17.6 mM and 80 µmol/m^2^/s. We anticipate that the presented methodology can be extended for short-term in vitro experiments to examine multiple conditions under a variety of parameters to accelerate the strain selection process.

## Figures and Tables

**Table 1:** Maximum growth rates (h^−1^) for *C*. *vulgaris* cells cultured in BG11 medium with different nitrate concentrations of 4.4, 8.8, and 17.6 mM for three days at 20 °C and different light intensities of 20, 80, and 200 µmol/m^2^/s using traditional batch cultures and a microfluidic device.

Light Intensity (µmol/m^2^/s)	Nitrogen Condition (mM)	Max. Growth Rate (per h)	
		Microfluidic Culture (mean/SD)	Traditional Batch Culture (×10^4^) (mean/SD)
20	4.4	0.10 ± 0.01	0.06 ± 0.002
	8.8	0.08 ± 0	0.12 ± 0.02
	17.6	0.05 ± 0.01	0.12 ± 0.006
80	4.4	0.10 ± 0.01	0.09 ± 0
	8.8	0.07 ± 0.04	0.05 ± 0.015
	17.6	0.27 ± 0.05	0.11 ± 0.009
200	4.4	0.06 ± 0.003	0.06 ± 0
	8.8	0.06 ± 0.01	0.05 ± 0.007
	17.6	0.17 ± 0.01	0.09 ± 0.01

## Supplementary Materials

The following are available online at https://www.mdpi.com/2218-273X/9/7/276/s1, Figure S1: Relationship between light over time represented from the data (A), and relation between uniform area (cm^2^) and light intensity (µmol/m^2^/s) (B). Where, (C) Relationship between light over time represented from Northern Illinois University data. http://www.vernier.com/innovate/inverse-square-law-light-experiment-mproved/. The consistency of light intensity over time was cleared. The lamp started with its highest power at zero time then its power was decreased until reached constant after 30 min. Presented resulted agreed with Richard Borne results. He suggested an improvement to the Inverse Square Law Light Experiment using our Optics Expansion Kit; his suggestion is to turn on the LED light sources for a half hour or more before starting data collection because the intensity of white LED lamp decreases somewhat over time when it is first turned on (http://www.vernier.com/innovate/inverse-square-law-light-experiment-improved/).
