# Supplementary material for: High-Throughput Screening of Chlorella Vulgaris Growth Kinetics inside a Droplet-Based Microfluidic Device under Irradiance and Nitrate Stress Conditions
Source: Biomolecules. 2019 Jul 12;9(7):276. doi: 10.3390/biom9070276 (PMC6681396; doi:10.3390/biom9070276)
Supplement: Supplementary file 1 [file biomolecules-09-00276-s001.pdf]

Electronic Supplementary Information

High-Throughput Screening of *Chlorella Vulgaris* Growth kinetics inside a Droplet-Based Microfluidic Device under Irradiance and Nitrate Stress Conditions

Marwa Gamal Saad [1, 2, *], Noura Sayed Dosoky [3, *], Muhammad Shuja Khan [4], Mohamed Shafick Zoromba [5, 6], Laila Mekki [7], Magdy El-Bana [2], David Nobles [8], and Hesham Mohamed Shafik [2]

[1] Department of Electrical and Computer Engineering, Texas A&M University, College Station, TX, USA ; marwa.aly@sci.psu.edu.eg
[2] Department of Botany, Faculty of Science, Port Said University, Port Said 42521, Egypt; heshamshafik@yahoo.co.uk (H.M.S.), and magdyelbana@sci.psu.edu.eg (M.E.)
[3] Department of Chemistry, University of Alabama in Huntsville, Huntsville, AL 35899, USA; nouradosoky@gmail.com
[4] Department of Electrical and Computer Engineering, University of Alabama in Huntsville, Huntsville, AL 35899, USA; m.khan@utah.edu
[5] Chemical and Materials Engineering Department, King Abdulaziz University, Rabigh 21911, Saudi Arabia; mohamedzoromba@yahoo.com
[6] Department of Chemistry, Faculty of Science, Port-Said University, Port Said 42521, Egypt; mohamedzoromba@yahoo.com
[7] Department of Botany, Faculty of Science, Suez Canal University, Ismailia, Egypt; lhmmekki_dr@hotmail.com
[8] UTEX Culture Collection of Algae, College of Natural Sciences, University of Texas at Austin, Austin, Texas, USA; dnobles@austin.utexas.edu

* Correspondence: marwa.aly@sci.psu.edu.eg; Tel.: +1-979-985-8091 (M.G.S.) and nouradosoky@gmail.com; Tel.: +1-256-457-0135 (N.S.D.)

Abstract: Biodiesel is an eco-friendly renewable fuel that can be derived from microalgae. Maximization of biomass and lipid productivities are considered the main challenges for algal biodiesel production. Since, conventional batch cultures are time-, space-, and reagents-consuming with many restrictions to apply many replicates, microfluidic technology has recently been emerged as an alternative low-cost and efficient technology with high throughput repeatability and reproducibility. Different applications of microfluidic devices in algal biotechnology have been reported including cell identification, sorting, trapping, and metabolic screening. In this work, Chlorella vulgaris was investigated by encapsulating in a simple droplet-based micro-array device at different light intensities of 20, 80, and 200 µmol/m^2/s combined with different nitrate concentrations of 17.6, 8.8, and 4.4 mM $NaNO_3$/L. The growth results for C. vulgaris within microfluidic device were compared to conventional batch culture method. In addition, the effect of combined stress of deficiencies in irradiance and nitrogen availability were studied to illustrate their impact on the metabolic profiling of microalgae. The results showed that the most optimum favorable culturing conditions for Chlorella vulgaris growth within the microfluidic channels were 17.6 mM $NaNO_3$/L and 80 µmol/m^2/s.

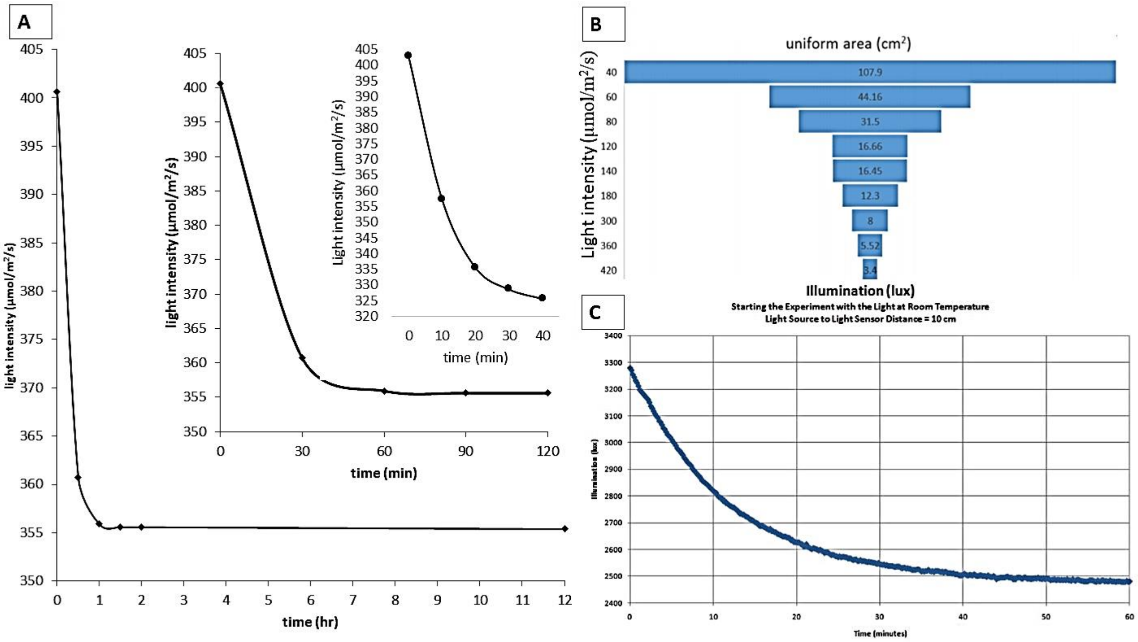

Figure S1. Relationship between light over time represented from our data (A), and relation between uniform area (cm²) and light intensity (μmol/m²/s) (B). Where, (C) Relationship between light over time represented from Northern Illinois University data. http://www.vernier.com/innovate/inverse-square-law-light-experiment- mproved/. The consistency of light intensity over time was cleared. The lamp started with its highest power at zero time then its power was decreased until reached constant after 30 min. presented resulted agreed with Richard Borne results. He was a researcher at Northern Illinois University. He suggested an improvement to the Inverse Square Law Light Experiment using our Optics Expansion Kit; his suggestion is to turn on the LED light sources for a half hour or more before starting data collection because the intensity of white LED lamp decreases somewhat over time when it is first turned on (http://www.vernier.com/innovate/inverse-square-law-light-experiment- improved/).
